# The value of consciousness: experiences worth having

**DOI:** 10.1098/rstb.2024.0303

**Published:** 2025-11-13

**Authors:** Léa Moncoucy, Krzysztof Dołęga, Catherine Tallon-Baudry, Axel Cleeremans

**Affiliations:** ^1^ Center for Research in Cognition and Neuroscience, Université Libre de Bruxelles Faculté des Sciences psychologiques et de l’éducation, Bruxelles, Belgium; ^2^ INSERM-ENS, Cognitive Neuroscience Laboratory, Paris, France

**Keywords:** phenomenal consciousness, subjective experience, adaptive signalling, intrinsic value, evolution

## Abstract

DC. Dennett (2020,personal communication) asked: ‘How do we go from doing things for reasons to having reasons for doing things?’. This question targets a fundamental shift in nature: while all organisms act in the way they do for reasons that are shaped by extrinsic evolutionary cost functions, some also act for reasons of their own, even engaging in behaviour that may be detrimental to their own existence. For such organisms, we argue, phenomenal experience—what it feels like—has intrinsic value. Here, we elaborate on the perspective developed by Axel Cleeremans and Catherine Tallon-Baudry (Cleeremans, Tallon-Baudry 2022 *Neurosci. Conscious*, **2022**, niac007. (doi:10.1093/nc/niac007)) and defend the claim that phenomenal experience broadens an organism’s ability to act in a manner that is not merely responsive to the objective value of an extrinsic evolutionary cost function but is also shaped by the preference-driven subjective value associated with items, situations, events or other agents. Importantly, we argue that the intrinsic value of subjective experience cannot always be reduced to other forms of extrinsic values, because subjective value can act not only as a driver of behaviour, but also as a target for behaviour.

This article is part of the theme issue ‘Evolutionary functions of consciousness’.

## 1. Introduction

Everything we do is directly influenced by how things *seem* to us. We eat foods that we like and avoid those we do not, we spend time with people whose company we enjoy and tend to avoid those whose company bothers or bores us, and so on. In other words, how we act is largely determined by how we *experience* things, by how things *feel* to us. Yet, why exactly do our experiences have such import? Why does it *matter* to us what Marmite or honey taste like? Or, even more broadly, what difference does it make for an organism to feel things? Answering this question amounts to finding a function for conscious experience, no doubt one of the core challenges faced by consciousness science today—for if we cannot find out what consciousness is good for, there is little hope of understanding its mechanisms. Here, taking Cleeremans & Tallon-Baudry’s [1] proposal as a starting point, we sketch an analysis of this issue, appealing to conceptual and evolutionary arguments as well as to empirical evidence.

Before we begin developing our case, it is essential to be clear about what we mean by conscious experience. In what follows, we will use ‘consciousness’ to refer specifically to *phenomenal* consciousness: what it subjectively feels like to have an experience of, say, the colour purple, the sound of waves, the taste of honey or the excruciating pain of having one’s leg crushed by a boulder. Importantly, we take it that phenomenal consciousness differs from mere sensitivity to distinct states of affairs. Smoke detectors, thermostats, plants and algorithms can all be appropriately described as being sensitive (and even reactive) to certain states of affairs, but these systems do not have *experiences* of any kind. There is nothing ‘it-is-like’ to be a mushroom, a plant or an algorithm.

From our perspective, there are two reasons why it is improbable that thermostats or large language models are subjects of conscious experience. First, we assume that, unlike mere sensitivity, phenomenal consciousness must involve some kind of *meta-sensitivity* to one’s own first-order sensitivities or capacities; what some authors have described as a form of ‘inner awareness’ [2–5]. Second, such meta-sensitivity is precisely what allows for a conscious system to stand in some relation to its own first-order sensations [6]. A camera, despite having a lens that is sensitive to light in ways not wholly unlike a human eye, does not *evaluate, appraise or care* about what it records, and a thermostat’s measure does not depend on whether the temperature is pleasant *to the thermostat*. Similarly, a simple reinforcement agent might be able to maximize its total reward over time, whether it is fitness or some arbitrary function. However, inasmuch as we can call maximizing such a function its *goal* or *purpose*, the values of such a function do not have deeper significance for the agent outside of the responses they trigger; they are only valuable *instrumentally*, but do not have any *intrinsic* value in themselves. Such systems register what states they are in, but they do not care about the value of such states in the same way that humans or other sentient creatures do.

By contrast, a subjective experience matters to the individual who experiences it. Humans and other sentient animals not only care about what they sense but tend to form preferences with respect to what experiences they would like to have in the future. Organisms that have the ability to feel attribute a subjective value to what they sense and seem to do so constantly and automatically [7]. Valence—the positive or negative tone of an experience—is not only the primary motivator of behaviour but also constitutes the most basic form of intrinsic value by which different experiences can be compared in decision-making [8]. While each experience has the potential to be allocated a positive and/or negative valence, the question whether all conscious experiences are valenced is an issue of ongoing empirical investigation [9,10]. Here, we follow Cleeremans & Tallon-Baudry [1] in positing that phenomenal experience is intrinsically valuable to the conscious subject. It is precisely in the intrinsic value associated with phenomenal states that we see a way of addressing the *why* question of consciousness. In the remainder of this introduction, we offer a brief preview of the core arguments we develop in the rest of this article.

We begin building our hypothesis about the potential function(s) of consciousness with a simple question: why are sensations worth caring for? Or, to paraphrase: what potential benefit could there be for an organism to care about their own sensations? While there are many ways in which one could try to answer these questions, adopting a naturalistic perspective suggests considering evolution as one of the possible answers. At least some sensations are likely to correlate with or be directly caused by conditions that will either increase or decrease an organism’s fitness, such as, for example, the presence of food, of viable mates, or of predators. This lends plausiblility to the assumption that sensations can carry information about the adaptive value of external states of affairs as well as of internal states of the body. Being receptive to this information, even in an imperfect, implicit and approximate manner, would then itself constitute an adaptive trait. To put it simply: sensations may be worth caring for because they can communicate something about the conditions that are hostile or beneficial to the organism.

This rather straightforward insight informs our first claim about the function of conscious experience, namely that most basic and paradigmatic forms of phenomenal states associated with sentience [11], such as the valenced feelings of core affect [12], have probably been selected for their capacity to serve as indicators of adaptive value. Being sensitive to the potential harms and benefits of different bodily and environmental conditions has not only enabled the emergence of more refined forms of behavioural control based on the immediate or potential adaptive values associated with the consequences of actions, but has also kicked open the doors for the appearance of more complex forms of learning and decision-making, from affective priming, categorization [13] and instrumental conditioning [14–16] to unlimited associative learning [17].^1^

Thus, our second claim about the function of consciousness is that the information about the adaptive value carried by phenomenal states plays the role of input to a number of more complex cognitive functions, which in turn makes it possible to control behaviour in increasingly more flexible ways. A prime example of how the availability of phenomenal experience can enable behavioural flexibility is that organisms with access to affective signals about the approximate adaptive value of different outcomes can engage in temporal and motivational trade-offs based on the valence associated with different actions [20,21]. For example, humans can regulate their homeostatic needs by counterbalancing unpleasant sensations (e.g. increasing physical effort to keep warm in a cold environment). Likewise, rats have been shown to willingly exchange the sensation of immediate discomfort (e.g. cold) for a later feeling of pleasure (e.g. tastier food, see [22]). An essential point here is to note that the relevant adaptive values are *extrinsic* to the organisms, who do not have direct access to these values in themselves. Rather, those values are signalled to them by proxy in the subjective valence of their sensations.

This brings us to the last and perhaps key claim we want to make about the function of consciousness, namely that once phenomenal experience plays the role of indicating the extrinsic value of different courses of action, it can acquire *intrinsic* value of its own. Consciousness—feeling things—begins to matter in and of itself to an organism. Such an organism can then begin seeking experiences for their own sake, that is, independently of the behaviours or outcomes that they were originally correlated with or caused by. As Mitchell [23] puts it, at that point, agents become ‘loci of concern’—they care about what they feel, and in caring about phenomenal states *in themselves*, they can end up decorrelating such states from their adaptive value. In this sense, consciousness can be both a functional adaptation shaped by natural selection and a kind of runaway process that enabled humans and other animals to at least partially free themselves from the shackles of evolution. This is made plain by the fact that humans routinely engage in endeavours that not only fail to increase their fitness (in the sense of natural selection) but often actually decrease it. Why is this the case? Why do we bungee jumping, run ultramarathons or just eat unhealthy foods and binge-watch TV shows? Well—for the thrill of it! That is, because such activities just *feel good*; and so, phenomenal states, initially functioning primarily as approximate, indirect measures of fitness, eventually became targets in and of themselves and have hence largely ceased to function as measures of extrinsic value.

We elaborate on and defend each of these points in the next three sections of this article. Importantly, our claims about the role of valenced conscious experience in shaping behaviour and cognition primarily concern the case of human experience, even though our shared phylogenetic history with other animals presumably offers a way of extending the same rationale to many non-human species. Our caution is motivated by the inherent difficulty of ensuring that any non-human species has phenomenal experience [24,25]. Furthermore, we are cognizant of the possibility that any of the cognitive processes we describe as conditional on phenomenal consciousness could, in principle, be implemented without it, either in some non-human species or in phenomenal zombies [26]. However, we take it that such possibilities would either require a world with an evolutionary history different from ours (i.e. one in which the capacities in question evolved along paths different from the ones by which they came to be in our actual world), or that they are a matter of purely conceptual speculation (as is the case with phenomenal zombies) [27]. In other words, we take the possibility of such non-phenomenal implementations as a logical rather than a nomological or temporal possibility.

## 2. Phenomenal consciousness as a proxy system for signalling evolutionary fitness

In modern evolutionary theory and population genetics, *fitness* is understood as an abstracted representation of an organism’s reproductive success and is usually defined as the average contribution to the next generation’s gene pool. Measures of fitness can be specified with respect to genotypes as well as phenotypes and can be made relative to particular environments or time spans. Still, fitness itself is usually considered to be a biologically objective property. Phenotypic traits, i.e. the observable and measurable characteristics of an organism, such as eye colour, type of plumage or shell shape, which are determined by genes and the environment, are considered to be *adaptive* when they enhance an organism’s probability of survival and reproduction [28]. Thus, in as much as survival contributes to an individual’s reproductive success, adaptive traits are usually considered to be fitness enhancing.^2^ Similarly, behaviours can also be considered adaptive or maladaptive, depending on whether they increase or decrease an organism’s chance for survival and reproduction. Common examples of adaptive behaviours in behavioural ecology include defending one’s territory and resources from rivals or engaging in mating rituals aimed at selecting the healthiest sexual mating partner [30]. In what follows, we will refer to *adaptive value* as a close, but imperfect indicator of whether a trait or behaviour is fitness enhancing.

Central to our argument is the fact that no organism has direct epistemic access to its fitness. In other words, no individual can objectively compute how fit they are, since their fitness depends on factors that cannot be directly appraised by the organism, such as the size of the next generation’s gene pool, etc. [31].^3^ Similarly, individuals also do not have access to information about the objective adaptive value of their traits or behaviours [35], for this value depends on factors that may be wholly independent of the organism, such as the stability of the ecological niche to which a particular trait or behaviour has been tailored. Nevertheless, some adaptive traits can function as signals that indirectly communicate information about an individual’s (potential) fitness to other organisms that are sensitive to those messages. The size of a male peacock’s tail is often invoked as an example of a handicap that functions as a signal of a male individual’s fitness to his potential mates [36]. On the side of behaviour, erecting elaborate constructions and performing complicated mating dances, such as the ones that different species of bird-of-paradise have become known for, seem to play the same function. Importantly, though, these traits and behaviours are examples of signals that are indirect, approximate, and *public*. A peacock’s tail might carry information for the females (something akin to ‘I am strong and fertile’) and even potential predators (‘here’s a prey with an easily grabbable tail’), but does not communicate anything to the peacock himself.

In addition to public signals of fitness, there are also *internal* states that play the role of signalling and promoting adaptive behaviour. Importantly, these states are *private* and carry information *for* the organism in which they occur.

Basic affective states, such as thirst or hunger, are prime examples of internal adaptive signals. For example, thirst is commonly conceptualized as both an anticipatory signal carrying information about the potential mismatch between the current and the expected (or optimal) homeostatic state, as well as a signal conveying the fact that such a mismatch has already taken place [37]. Subjectively, though, signals of this type can also be defined in terms of their valence, i.e. the positive or negative hedonic tone that makes them feel, respectively, pleasant or unpleasant to the subject. The valenced aspect of these signals is also commonly assumed to be responsible for their motivational force. The feeling of thirst gets increasingly more unpleasant the longer one goes without sustenance, thus impressing more and more urgently on the organism the need to take action [37]. Conversely, the pleasure associated with ingesting sugar or partaking in sexual activity serves as a reward that is supposed to motivate seeking out similar activities in the future. Valenced states can, therefore, be described as fulfilling the function of signalling the approximate fitness *of both: the organism*, in virtue of carrying *descriptive* information about its current state (‘this bodily state is bad for you and staying in it might lead to death’); and of a *set of potential actions*, in virtue of having a *prescriptive* role of directing the organisms towards those actions that will take it out of the current state (‘find food, now!’).^4^^,5^

This idea is not fundamentally new, as similar proposals have already been developed with regard to pain [41,42]. However, the crucial point for us is that the information carried by positive or negative hedonic states is made available to the organism through the valenced aspect of these affective states. What we mean by this is that it is the very unpleasantness of hunger that informs the organism about its maladaptive state, and it is this very feeling that simultaneously impinges on it the importance of seeking food. Critically, having the capacity to entertain and be sensitive to such hedonic feelings is usually understood as a key criterion of animal sentience [43].

Thus, the core of our proposal is this: the probable evolutionary function of phenomenal experience, that is, the function for which it was originally selected, is to signal the approximate adaptive value of either internal or external states to the subject. The awfulness of physical injuries, the disgust upon smelling rotten food, but also the sweetness of sugar and the pleasure of courtship and copulation all indirectly communicate the approximate adaptive value of certain bodily states and of the actions they are associated with. This idea not only has an important historical precursor in James’ [44] views on consciousness, but also aligns with present day perspectives on the role of affective states in guiding decision-making and adaptive behaviour [18,45–49].

From the perspective of a sentient being, following such signals requires no inherent understanding of how visiting or avoiding particular states (or carrying out certain actions) will improve one’s survival or reproductive success.^6^ The positive and negative valences of basic affective states have been tailored and calibrated for their adaptive role over the evolutionary scale. Some things feel good to a particular organism and others feel bad to it because, on average and over a timespan of generations, there was an adaptive advantage in doing the former and in avoiding the latter. Nevertheless, just like in the case of the evolution of public adaptive signalling, the private signals of phenomenal experience could not play their adaptive role without being appropriately received by other internal systems. In other words, the evolution of such signals must have been simultaneous with the co-evolution of *consumer systems* (in the sense used by [51]) that are sensitive to the information phenomenal experience conveys. It is precisely because sentient beings have been outfitted with both adaptive internal value signalling systems and with systems capable of decoding (or interpreting) the information of those signals that they can properly relate to those signals and *care* about their contents.

The existence of sentience is at the root of the difference between affective *feelings* and mere behavioural *reactions*, such as the distinction between pain and nociception. It is widely known that in many cases, the feeling of pain occurs *after* the behavioural reaction to the stimulus responsible for it. For example, the nociceptive reflex of jerking one’s hand away from a hot stove occurs *before* the burning feeling of pain is experienced consciously. The jerking response is hard-wired, i.e. it is an innate behaviour performed in response to a certain type of stimulus. It is automatic, does not have to be learned, cannot be unlearned (though it can, in some cases, be inhibited) and is common to the whole species. The nociceptive reflex is an example of an adaptive trait, but what makes it different from the experience of pain? Our answer is simple: on its own, nociception lacks the information that tells other cognitive systems why it should not be repeated. If the reflex was not followed by the experience of pain, we would be unable to learn *why* we should not repeat the same behavioural loop again. We would not *care* about the stimulus or the sensation associated with the reflex. Indeed, subjects who are suffering from pain asymbolia [52] or have received anterior cingulotomy to alleviate chronic pain [53] tend to report that they do sense the location of their injuries ‘but that they no longer care’ about them [54, p. 663]. Furthermore, reported cases of pain asymbolic subjects show that they do not signal distress, express wishes for the cessation of noxious stimuli, nor do they display typical reactions aimed at self-preservation [55], even when the cause of the pain could inflict lasting damage to the body. Therefore, it seems that what makes pain adaptive is not just nociception, but rather the valenced aspect of the associated conscious experience, which motivates one to act in a way that will stop the feeling and allows for the formation of generalizations regarding the effects of potentially dissimilar stimuli. For pain to play its adaptive role, it is not enough to merely detect or automatically react to tissue damage. One must also have a valenced experience that will motivate the feeling organism to care about the long-term preservation of their own body as well as allow them to recognize and avoid potentially harmful stimuli in the future. In this respect, it is significant that pain asymbolia is typically brought about not by abnormalities in the peripheral nervous system or in the detection of noxious stimuli, but by changes in the processing of core affective signals, typically caused either by lesions between the cingulate gyrus and the insular cortex, or by pharmacological interventions targeting the opioidergic pathways responsible for modulating the feeling of pleasure and pain. Thus, the difference between mere sensation or reaction and proper experience is in the effects that it has on the downstream processes. In the next section, we speculate about the nature of such processes and on why certain types of consumer systems may be necessary, but not sufficient, for consciousness to arise.

## 3. Phenomenal consciousness as the locus of adaptive control

In the preceding section, we have argued that the function of phenomenal experience is to act as a proxy signal for the adaptive value of different kinds of internal and external states. We have also pointed out that such signals can only fulfil this function if they are coupled to consumer systems that are sensitive to the information they convey. Here, we want to suggest that internal signals only *become* phenomenal signals in virtue of serving as inputs to particular kinds of co-evolved systems or processes. That is, such signals do not feel like anything unless they are picked up and processed by the appropriate consumer systems, as illustrated by the case of pain asymbolia. However, what exactly are those consumer systems or processes?

As we mentioned before, bodily pain and pleasure are usually assumed to be at the heart of most basic forms of sentience, and therefore, of phenomenal experience [24]. These kinds of experiences are clearly there to promote visiting certain bodily states and avoiding certain other states; hence, they are likely to have evolved in order to facilitate behavioural control. For example, a typical reaction to pain that is expected from a sentient being is to act in a way that will either remove itself from the perceived immediate source of pain and/or seek relief from the pain (either by engaging in some protective and self-grooming behaviour or by self-administering analgesics). Hence, it is likely that the most basic forms of consumer systems that have co-evolved with pain and pleasure signals are those involved in the integration of sensory/proprioceptive and affective information and in making that information available for immediate behavioural control [56]. Notice, however, that merely escaping an occurrent painful state is different from actively avoiding visiting such states in the first place. The latter ability requires the capacity to recognize, learn, memorize and finally anticipate the kinds of stimuli that have previously caused or that have at least correlated with painful experiences (see also Crystal [57]). Therefore, more advanced kinds of adaptive behaviour, such as prospective avoidance of harm or engaging in motivational trade-offs require more sophisticated systems allowing for the sensitivity, processing and storage of the available information about the adaptive value of bodily and environmental states.

Jablonka & Ginsburg [17, p. 414] provide a comprehensive list of neurophysiological and cognitive mechanisms that they stipulate to be the markers of an evolutionary transition from unconscious to conscious biological systems. We agree with them that systems responsible (i) for the discrimination and differentiation of composite and complex stimuli, (ii) for the sequential as well as asynchronous integration of information, storage, and (iii) for the maintenance of this information in working memory, (iv) for the evaluation or re-evaluation of this information, and finally, (v) for its use for the purpose of prioritization through selective attention and global availability, are probably all necessary for the emergence of consciousness. Presumably, these capacities did not evolve wholesale and all at once, but instead appeared in piecemeal fashion in response to different evolutionary pressures (see also Jablonka & Ginsburg [58]). Nevertheless, once they were in place and formed a network of reciprocally connected consumer systems, they brought about a new form of informational organization that enabled the manipulation, categorization, and re-description of sensory and proprioceptive signals for the purpose of improving the control of behaviour, but also, crucially, for the learning of preferences and the formation of stable goals. In the end, the ability to recognize, evaluate, store and compare stimuli with respect to the potential outcomes that they correlate with resulted in the kind of novel representational capacity that itself allowed for the appearance of a subjective point of view on the world. The presence of value-laden representations of sensory stimulations as well as potential goal states paved the road for the emergence of selfhood, first in the implicit form of a minimal self [59,60], and later in the form of an explicit self-representation [61]. The point we wish to highlight here is that valenced states *imply* the existence of a self—a system or collection of systems that give rise to the ‘locus of concern’—a subject of experience that minimally has an attitude towards the stimuli and conditions with which it interacts. Of course, while these evolutionary innovations probably took place multiple times and in slightly different guises across different branches of the phylogenetic tree, they ultimately led to the emergence of consciousness as we know it from our daily experience.

Although we do not want to commit ourselves to the fine details of one particular cognitive architecture, leaving the specification of the exact interactions and influences between the aforementioned cognitive capacities as an open empirical question, we specify below our position with respect to some existing proposals. First, as we have already made clear, we largely agree with the assumptions of Ginsburg & Jablonka’s [18] unlimited associative learning hypothesis. Second, our view is also compatible with the hedonic interface theory [47] according to which the primary function of consciousness is goal-directed behaviour that is primarily controlled by desires understood as cognitive representations of goal values revealed in hedonic experiences.

Nevertheless, our perspective also departs from these two views in some significant ways. The hedonic interface theory excludes all forms of instrumental learning from the set of conscious functions, whereas the unlimited associative learning hypothesis admits only ‘compound’ forms of such learning in which the conditional stimulus or the reinforced action ‘consist of several features or action-patterns that are learned only as a whole, rather than separately’ [62, p. 3]. However, a recent series of experiments by Skora and colleagues has rather decisively demonstrated that, at least in humans, this type of learning does require conscious awareness [14–16]. Importantly, the paradigm used in these studies required participants to be sensitive to only one feature of the stimulus (shape), rather than their combination (e.g. shape and colour or orientation). Hence, even though simple forms of instrumental learning might not use all the cognitive functions required for unlimited associative learning [17], we are inclined to include non-compound forms of such learning as belonging to the set of capacities that do depend on conscious experience. We conjecture that this is probably owing to the fact that affective rewards need to be felt in order to be fully used in controlling more complex and temporarily extended behaviours. This highlights another point of contention between our position and that of other authors. On the one hand, we disagree with Jablonka and Ginsburg that the source of intrinsic affective value is related to Panksepp’s basic emotion of seeking ([63]; cf. [17]), as we think that the subjective feeling of pleasure is a primary source of information about a goal’s desirability and the motivation to obtain it [64]. On the other hand, we remain unsure about one of the central assumptions of the hedonic interface hypothesis, which states that ‘the representation of goal value, although initially grounded on affective and hedonic experience, is in fact purely cognitively and affect free’ [47, p. 80]. Finally, even though we remain committed to the existence of a network of reciprocal connections that allow information to be integrated and shared between different localized processes, for the purposes of this article, we remain agnostic about the processes through which information becomes globally available [65] and the exact connectivity that allows for such availability (though see [6] for a suggestion about the relevance of higher-order processes). Notably, empirical evidence [66,67] suggests that it is conscious phenomenology that determines the type of attention that is deployed towards a visual stimulus rather than the other way around, casting conscious experience as the trigger of executive functions, rather than the other way around. Thus, as Ned Block writes, ‘it may be conscious phenomenology that promotes global broadcasting, something like the reverse of what the global workspace theory of consciousness supposes’ [68, p. 9].

## 4. Phenomenal consciousness as both a signal and a target

Thus far, we have suggested that phenomenal consciousness has the function of signalling the adaptive value of internal and external states to sentient beings, and that the felt qualities of such experiences depend on the kinds of cognitive systems sensitive to this adaptive information. However, a proponent of reductive naturalism could raise the objection that we have not yet shown that the phenomenal aspect of experience is necessary in accounting for the adaptive value of internal signals or in explaining the ways in which they impact behaviour. In other words, one could claim that the subjective aspect of experience is either redundant to our story because, hypothetically, the same kind of adaptive signalling could have been possible without producing phenomenal experience, or that phenomenal consciousness is a mere by-product of the neural structures’ functioning. Here, we will push against such objections by arguing that in fulfilling its signalling function, phenomenal experience can acquire significance of its own, transitioning from merely *guiding* behaviour to being its *target*.

If phenomenal experiences act as proxy signals of adaptive value, then this value is *extrinsic* to the cognitive consumer systems and to the subject, as it depends on factors that are not only independent of the experience itself, but can also be independent of the subject. However, we have been arguing that at least certain kinds of experiential states, such as hedonic pleasure and pain, were initially selected for the purpose of signalling adaptive value of certain states, by acting as inputs for different co-evolved evaluation and learning mechanisms that reinforce the behaviours associated with those states. It is through this coupling of signals and reinforcement systems that hedonic states can act as rewards and punishments for certain kinds of behaviours. Actions leading to extrinsically *valuable* outcomes are reinforced through the pleasurable experiences, and actions leading to extrinsically *detrimental* outcomes are reinforced through being unpleasant or outright painful. So, the signals of extrinsic adaptive value for the organisms became inherently motivating and thus *intrinsically* valuable to them as subjects. From the individual perspective of an organism, the hedonic motivation for the kinds of behaviours that are associated with positive adaptive states comes not from their objective benefit of fitness enhancement (which, as we argued, is inaccessible to the organism), but from the positive valence they elicit in the associated experiences. Simply put: we seek out experiences that are pleasant and tend to avoid those that are not (unless, of course, one is engaging in some form of motivational trade-off).

Although this view, or at least something close to it, seems to be quite widespread in the literature, this answer would be unlikely to satisfy our critics. After all, intrinsic value signalling could, at least in principle, be realized by unconscious systems. Hence, the appeal to experience as a vehicle of intrinsic value does not secure the explanatory role of experience as such. We agree, but we also want to go further. Thus far, we have demonstrated how phenomenal experiences have gone from serving as proxy signals for extrinsic use to playing the role of intrinsic evaluations that equip the cognitive system with self-contained motivational properties. However, to us, the crucial point is that this reinforcement process directs subjects to seek certain experiences for their *own* sake rather than solely as a means to an adaptive end. Thus, through this process, it is the experiences themselves that become the targets of behaviour, and certain forms of behaviour cannot be fully accounted for without invoking the subjective quality of the experiences they bring about. The reason for this is that at least some hedonic experiences that initially had the function of serving as approximate indicators of adaptive value can, under some circumstances, be decoupled from the objective fitness function and become an unreliable signal about the extrinsic value of whatever behaviour or outcome they are associated with. Thus, as experiences become targets of behaviour, their intrinsic value can be severed from their evolutionary function, misrepresenting the extrinsic value of (and yet still motivating) whatever activities that bring them about.

Consider, for example, the rewarding experience that we get from consuming sugary foods. It is plausible that we respond to sugar in the way we do because the experiences of pleasure and sweetness play the role of incentives (and/or reinforcers) for seeking out food items that provide a quick energy boost. Given the relatively high calorific needs associated with large brains and the kinds of energetically poor environments that our ancestors evolved in, it is not difficult to see how this kind of experiential incentive would promote adaptive behaviours. However, a trait that was beneficial in an energetically sparse environment—as was the case during the vast majority of our species’ evolutionary history—can become harmful in a highly industrialized one, where the (organized) overabundance of sugar-rich dietary options can lead not only to an overconsumption of sugar, but even to addiction. In such instances, the conscious experience, which is the primary motivator, becomes separated from its initial adaptive purpose, overriding survival concerns or even promoting outright maladaptive behaviours.

This intrinsic value of consciousness is further evident in behaviours observed across diverse species that do not appear to directly enhance fitness. Animals, including humans, often engage in activities driven by intrinsic motivation rather than strict survival needs, from masturbation to the arts, or even behaviours that entail risk without an apparent survival benefit such as BASE jumping. We argue that they engage in such actions because they find them to be intrinsically rewarding. In this view, consciousness not only guides survival-driven behaviour but also enables organisms to pursue goals shaped by the inherent value of conscious experiences. As these experiences become significant in their own right, consciousness can inspire behaviours that reflect another, additional, intrinsic purpose. Of course, we are aware that for any example of activity driven by intrinsic motivation we provide, it might be possible to find a potential *post hoc* evolutionary benefit (e.g. playing music might convey additional social benefits while risky behaviour might be an opportunity to signal physical prowess to potential mates). However, such benefits could also be examples of exaptations (traits that became relevant for functions different from the ones they were originally adapted or selected for) that have emerged *after*, or even *in response to*, the relevant behaviours have become valued and stabilized by the agents, as is the case for the feathers that are functionally relevant for flying, even if they first emerged for temperature regulation. Ultimately, such questions are empirical matters that should be settled through appropriate investigation. The point we make here is that the intrinsic value of conscious experiences constitutes a motivational drive to engage in activities, regardless of whether they might turn out to convey some evolutionary advantage over much longer timescales. In other words, the fact that phenomenal states have become the very targets of our behaviour is a drive for novel repertoires to emerge in the first place, with evolutionarily driven pressures coming in a second step.

Finally, the fact that conscious experiences have intrinsic value for their subjects speaks in favour of the idea that those experiences are not a mere by-product of regulating processes, but that they have a supplementary effect on the organism. The reason for this is simple: it is the subjective experiences themselves and not their underlying processes that usually serve as targets for the behaviours we engage in. In other words: when we pursue a pleasant experience, we are trying to (re)create sensations and feelings we expect to enjoy based on our past experiences, rather than aiming to stimulate our μ-opioid receptors, etc., (even though the former does depend on the latter, e.g. [69]). Pleasure, which evolved to signify and motivate organisms towards adaptive behaviours, becomes self-reinforcing to the point where, either the stimulus-behaviour loops it is supposed to motivate us towards become maladaptive (as in the case of overconsumption of sugar), or the experiences themselves become decoupled from the behaviours they were supposed to reinforce in lieu of other behaviours that produce the same experience but do not convey any clear adaptive advantage (as in the case of masturbation). Interestingly, this echoes Goodhart’s law in economics, which states that ‘Any observed statistical regularity will tend to collapse once pressure is placed upon it for control purposes’ [70, p. 116], or, in simpler terms: when a measure becomes a target, it ceases to be a good measure.

The aforementioned cases show that conscious states are not mere by-products because, in becoming the targets of behaviour, they are not treated as transparent to the subject, their significance no longer lies in what they represent (e.g. food items that increase adaptiveness and help to maintain homeostasis), but in themselves. The internal signals themselves, rather than what they represent or indicate, become the targets for the organism, implying that, at the very least, the organism must have some form of apprehension of the valence of the signals as opposed to what they are supposed to stand for or communicate to the subject.

## 5. Conclusion

Does consciousness play an evolutionary function, or is it merely a spandrel—an evolutionary by-product with no adaptive value? In the present article, we argue that it is both. On the one hand, we spelled out a proposal that phenomenal experience might initially have emerged as an adaptive trait, conveying information about the approximate adaptive value of different states and actions. On the other hand, we have also argued that, with enough behavioural flexibility and cognitive complexity, such signals could become decoupled from their evolutionary function and lead to forms of behaviour that are completely at odds with evolutionary ends. This duality suggests that consciousness is not simply an adaptive by-product, but a phenomenon that shapes both survival-driven and experience-driven behaviours: ‘a *fighter for ends*, of which many, but for its presence would not be ends at all’ [44, p.141]. Understanding consciousness requires recognizing its role as both an adaptive mechanism and a source of intrinsic value, with implications for the evolution of complex, flexible, and sometimes non-adaptive behaviours across the animal kingdom.

Thus, rather than treating consciousness as an example of a spandrel, it might be better described as a runaway process. What we mean by this is that conscious experience has probably been a product of (either direct or indirect) evolutionary selection at some point in time. However, in the same way that some biologists, including Darwin, have claimed that sexual selection might have become a separate process from natural selection (e.g. [71]), the very characteristics that have made conscious experience adaptive (i.e. value signalling and reinforcement) would, under certain circumstances, lead it to become a completely novel arena of behavioural valuation (see also Jablonka & Ginsburg, [58]). On this picture, consciousness would allow for new behaviours and new kinds of behavioural control to emerge and persist independently of, and sometimes in spite of, their adaptive value. Although this perspective is merely a hypothesis in need of further work and support, it presents consciousness as a phenomenon that is both biological in origin, and transcends the evolutionary laws that shaped its development, offering a potential to explain our intuitions that subjective experience is unlike any other natural phenomenon.

## Data Availability

This article has no additional data.

## Footnotes

^1^
*Unlimited associative learning* is an open and domain-general form of associative learning that has been proposed as a potential evolutionary origin of consciousness by Ginsburg & Jablonka [18]. What separates it from more basic forms of learning are the following features: sensitivity to novel and/or compound stimuli, capacity for trace conditioning and second-order conditioning, as well as the ability to form flexible and malleable associations with value [19, p. 56].

^2^
It is important to note, that fitness and adaptiveness are distinct properties that can, at times, dissociate. Some adaptations may increase the chances of organisms’ survival in a particular ecological niche without improving their objective fitness. A common example of such dissociation comes from Dobzhansky [29], who pointed out that Californian redwoods (*Sequoia sempervirens*) are highly adapted to their niche, but are in danger of extinction, owing to environmental changes and the gradual disappearance of their habitat.

^3^
Another, more serious, problem is that fitness is often defined as a property of populations or traits and not individuals [32,33], though some authors have pushed against this characterization [34]. Here, we do not wish to take a hard stance on the correct interpretation of fitness.

^4^
In other words, these signals might function as what Millikan [38] dubbed ‘pushmi-pullyou’ representations, a suggestion that has been previously made by Martínez [39].

^5^
Here, we do not want to take a stance on the exact nature of the contents of these representations, though the general view which emerges from the present article is perhaps closest to the one espoused by Barlassina & Hayward [40].

^6^
This is an example of what Dennett labelled as ‘competence without comprehension’ [50].

## References

[1] Cleeremans A, Tallon-Baudry C. 2022 Consciousness matters: phenomenal experience has functional value. Neurosci. Conscious. **2022**, c007. (10.1093/nc/niac007) PMC9036654 35479522

[2] Armstrong D. 1968 A materialist theory of the mind. London, UK: Routledge.

[3] Lycan W. 1996 Consciousness and experience. Cambridge, MA: MIT Press.

[4] Rosenthal DM. 2005 Consciousness and mind. Oxford, UK: Oxford University Press.

[5] Rosenthal DM. 2012 Awareness and identification of self. In Consciousness and the self: new essays (eds L JeeLoo, J Perry), pp. 22–50. Cambridge, UK: Cambridge University Press.

[6] Cleeremans A, Achoui D, Beauny A, Keuninckx L, Martin JR, Muñoz-Moldes S, Vuillaume L, de Heering A. 2020 Learning to be conscious. Trends Cogn. Sci. **24**, 112–123. (10.1016/j.tics.2019.11.011) 31892458

[7] Lebreton M, Jorge S, Michel V, Thirion B, Pessiglione M. 2009 An automatic valuation system in the human brain: evidence from functional neuroimaging. Neuron **64**, 431–439. (10.1016/j.neuron.2009.09.040) 19914190

[8] Shenhav A. 2024 The affective gradient hypothesis: an affect-centered account of motivated behavior. Trends Cogn. Sci. **28**, 1089–1104. (10.1016/j.tics.2024.08.003) 39322489 PMC11620945

[9] Lebrecht S, Bar M, Barrett LF, Tarr MJ. 2012 Micro-valences: perceiving affective valence in everyday objects. Front. Psychol. **3**, 107. (10.3389/fpsyg.2012.00107) 22529828 PMC3328080

[10] Mentec I, Ivanchei II, Cleeremans A. 2024 Exploring the role of valence in conscious perception: insights from similarity judgments and deep learning models. PsyArXiv. (10.31234/osf.io/j5mdk) PMC12941196 41767289

[11] Birch J, Schnell AK, Clayton NS. 2020 Dimensions of animal consciousness. Trends Cogn. Sci. **24**, 789–801. (10.1016/j.tics.2020.07.007) 32830051 PMC7116194

[12] Barrett LF, Bliss-Moreau E. 2009 Affect as a psychological primitive. Adv. Exp. Soc. Psychol. **41**, 167–218. (10.1016/S0065-2601(08)00404-8) 20552040 PMC2884406

[13] Lähteenmäki M, Hyönä J, Koivisto M, Nummenmaa L. 2015 Affective processing requires awareness. J. Exp. Psychol. **144**, 339–365. (10.1037/xge0000040) 25559654

[14] Skora LI, Yeomans MR, Crombag HS, Scott RB. 2021 Evidence that instrumental conditioning requires conscious awareness in humans. Cognition **208**, 104546. (10.1016/j.cognition.2020.104546) 33360281

[15] Skora LI, Livermore JJ, Nisini F, Scott RB. 2022 Awareness is required for autonomic performance monitoring in instrumental learning: evidence from cardiac activity. Psychophysiology **59**, e14047. (10.1111/psyp.14047) 35304762 PMC9541215

[16] Skora LI, Livermore JJ, Dienes Z, Seth AK, Scott RB. 2023 Feasibility of unconscious instrumental conditioning: a registered replication. Cortex **159**, 101–117. (10.1016/j.cortex.2022.12.003) 36621202

[17] Jablonka E, Ginsburg S. 2022 Learning and the evolution of conscious agents. Biosemiotics **15**, 401–437. (10.1007/s12304-022-09501-y)

[18] Ginsburg S, Jablonka E. 2019 The evolution of the sensitive soul: learning and the origins of consciousness. Cambridge, MA: MIT Press.

[19] Birch J, Ginsburg S, Jablonka E. 2020 Unlimited associative Learning and the origins of consciousness: a primer and some predictions. Biol. Philos. **35**, 36. (10.1007/s10539-020-09772-0) PMC7116763 33597791

[20] De Martino B, Cortese A. 2023 Goals, usefulness and abstraction in value-based choice. Trends Cogn. Sci. **27**, 65–80. (10.1016/j.tics.2022.11.001) 36446707

[21] Brown S, Birch J. 2025 When and why are motivational trade-offs evidence of sentience? Phil. Trans. R. Soc. B **380**, 20240309. (10.1098/rstb.2024.0309) 41229290 PMC12612706

[22] Cabanac M. 1992 Pleasure: the common currency. J. Theor. Biol. **155**, 173–200. (10.1016/s0022-5193(05)80594-6) 12240693

[23] Mitchell KJ. 2023 Free agents. how evolution gave us free will. Princeton, NJ: Princeton University Press.

[24] Birch J, Burn C, Schnell A, Browning H, Crump A. 2021 Review of the evidence of sentience in cephalopod molluscs and decapod crustaceans. London, UK: LSE Consulting. LSE Enterprise Ltd. See https://www.lse.ac.uk/News/News-Assets/PDFs/2021/Sentience-in-Cephalopod-Molluscs-and-Decapod-Crustaceans-Final-Report-November-2021.pdf.

[25] Browning H, Birch J. 2022 Animal sentience. Philos. Compass **17**, e12822. (10.1111/phc3.12822) 35859762 PMC9285591

[26] Chalmers D. 1996 The conscious mind: in search of a fundamental theory. Oxford, UK: Oxford University Press.

[27] Dennett DC. 1995 The unimagined preposterousness of zombies. J. Conscious. Stud. **2**, 322–326.

[28] Dobzhansky T. 1956 Genetics of natural populations. XXV. Genetic changes in populations of Drosophila pseudoobscura and Drosophila persimilis in some localities in California. Evolution **10**, 82–92. (10.1111/j.1558-5646.1956.tb02832.x)

[29] Dobzhansky T. 1968 On some fundamental concepts of Darwinian biology. In Evolutionary biology (eds T Dobzhansky, MK Hecht, WC Steere), pp. 1–34. Boston, MA: Springer. (10.1007/978-1-4684-8094-8_1)

[30] Staddon JER. 1983 Adaptive behavior and learning. Cambridge, UK: Cambridge University Press.

[31] Wadgymar SM, Sheth S, Josephs E, DeMarche M, Anderson J. 2024 Defining fitness in evolutionary ecology. Int. J. Plant Sci. **185**, 000–000. (10.1086/729360) PMC11257499 39035046

[32] Dawkins R. 1976 The selfish gene. London, UK: Best Books.

[33] Sober E. 2013 Trait fitness is not a propensity, but fitness variation is. Stud. Hist. Philos. Biol. Biomed. Sci. **44**, 336–341. (10.1016/j.shpsc.2013.03.002) 23591050

[34] Pence C, Ramsey G. 2013 A new foundation for the propensity interpretation of fitness. Br. J. Philos. Sci. **64**, 851–881. (10.1093/bjps/axs037)

[35] van Hateren JH. 2017 A unifying theory of biological function. Biol. Theory **12**, 112–126. (10.1007/s13752-017-0261-y) 28680371 PMC5487967

[36] Grafen A. 1990 Biological signals as handicaps. J. Theor. Biol. **144**, 517–546. (10.1016/s0022-5193(05)80088-8) 2402153

[37] Leib DE, Zimmerman CH, Knight ZA. 2016 Thirst. Curr. Biol. **26**, R1247–R1271. (10.1016/j.cub.2016.11.019) PMC5957508 27997832

[38] Millikan RG. 1995 Pushmi-pullyu representations. Philos. Perspect. **9**, 185–200.

[39] Martínez M. 2011 Imperative content and the painfulness of pain. Phenomenol. Cogn. Sci. **10**, 67–90. (10.1007/s11097-010-9172-0)

[40] Barlassina L, Hayward MK. 2019 More of me! less of me!: reflexive imperativism about affective phenomenal character. Mind **128**, 1013–1044. (10.1093/mind/fzz035)

[41] Kozuch B. 2020 No pain, no gain (in Darwinian fitness): a representational account of affective experience. Erkenntnis **85**, 693–714. (10.1007/s10670-018-0044-2)

[42] Kolodny O, Moyal R, Edelman S. 2021 A possible evolutionary function of phenomenal conscious experience of pain. Neurosci. Conscious. **2021**, niab012. (10.1093/nc/niab012) 34141452 PMC8206511

[43] Broom DM. 2022 Concepts and interrelationships of awareness, consciousness, sentience, and welfare. J. Conscious. Stud. **29**, 129–149. (10.53765/20512201.29.3.129)

[44] James W. 1890 The principles of psychology. New York, NY: Holt.

[45] Bechara A, Damasio AR. 2005 The somatic marker hypothesis: a neural theory of economic decision. Games Econ. Behav. **52**, 336–372.

[46] Burgdorf J, Panksepp J. 2006 The neurobiology of positive emotions. Neurosci. Biobehav. Rev. **30**, 173–187. (10.1016/j.neubiorev.2005.06.001) 16099508

[47] Dickinson A, Balleine B. 2010 Hedonics: the cognitive–motivational interface. In Pleasures of the brain (eds ML Kringelbach, AC Berridge), pp. 74–84. New York, NY: Oxford University Press.

[48] Mendl M, Paul ES. 2020 Animal affect and decision-making. Neurosci. Biobehav. Rev. **112**, 144–163. (10.1016/j.neubiorev.2020.01.025) 31991192

[49] Veit W. 2023 Complexity and the evolution of consciousness. Biol. Theory **18**, 175–190. (10.1007/s13752-022-00407-z)

[50] Dennett DC. 2017 From bacteria to Bach and back: the evolution of minds. New York, NY: W. W. Norton & Company.

[51] Millikan R. 1984 Language, thought and other biological categories. Cambridge, MA: MIT Press.

[52] Berthier M, Starkstein S, Leiguarda R. 1988 Asymbolia for pain: a sensory-limbic disconnection syndrome. Ann. Neurol. **24**, 41–49. (10.1002/ana.410240109) 3415199

[53] Wilkinson HA, Davidson K, Davidson RI. 1999 Bilateral anterior cingulotomy for chronic noncancer pain. Neurosurgery **47**, 1129–1136. (10.1227/00006123-199709000-00100) 10549929

[54] Carruthers P. 2018 Valence and value. Philos. Phenomenol. Res. **97**, 658–680. (10.1111/phpr.12395)

[55] Schilder P, Stengel E. 1931 Asymbolia for pain. Arch. Neurol. Psych. **25**, 598–600. (10.1001/archneurpsyc.1931.02230030156007)

[56] Klein C, Barron AB. 2025 Phenomenal interface theory: a model for basal consciousness. Phil. Trans. R. Soc. B **380**, 20240301. (10.1098/rstb.2024.0301) 41229298 PMC12612696

[57] Crystal JD. 2025 Episodic memory in non-humans: an approach to understand an evolutionary function of consciousness. Phil. Trans. R. Soc. B **380**, 20240304. (10.1098/rstb.2024.0304) 41229289 PMC12612702

[58] Jablonka E, Ginsburg S. 2025 Consciousness: its goals, its functions and the emergence of a new category of selection. Phil. Trans. R. Soc. B **380**, 20240310. (10.1098/rstb.2024.0310) 41229292 PMC12612698

[59] Zahavi D. 2005 Subjectivity and selfhood: investigating the first-person perspective. Cambridge, MA: MIT Press.

[60] Park HD, Tallon-Baudry C. 2014 The neural subjective frame: from bodily signals to perceptual consciousness. Phil. Trans. R. Soc. B **369**, 20130208. (10.1098/rstb.2013.0208) 24639580 PMC3965163

[61] Metzinger T. 2008 Empirical perspectives from the self-model theory of subjectivity: a brief summary with examples. In Progress in brain research, vol. 168 (eds B Rahul, KC Bikas), pp. 215–246. Amsterdam, The Netherlands: Elsevier. 10.1016/S0079-6123(07)68018-2 18166398

[62] Bronfman ZZ, Ginsburg S, Jablonka E. 2016 The transition to minimal consciousness through the evolution of associative learning. Front. Psychol. **7**, 1954. (10.3389/fpsyg.2016.01954) 28066282 PMC5177968

[63] Panksepp J. 2011 Cross-species affective neuroscience decoding of the primal affective experiences of humans and related animals. PLoS One **6**, e21236. (10.1371/journal.pone.0021236) 21915252 PMC3168430

[64] Moncoucy L, Dolega K. Unknown pleasures: do we have evidence for unconscious, positively valenced hedonic states? SSRN 19–23. (10.2139/ssrn.5296974)

[65] Dehaene S, Changeux JP. 2011 Experimental and theoretical approaches to conscious processing. Neuron **70**, 201–227. (10.1016/j.neuron.2011.03.018) 21521609

[66] Hsu SM, George N, Wyart V, Tallon-Baudry C. 2011 Voluntary and involuntary spatial attentions interact differently with awareness. Neuropsychologia **49**, 2465–2474. (10.1016/j.neuropsychologia.2011.04.024) 21569785

[67] Tallon-Baudry C, Campana F, Park HD, Babo-Rebelo M. 2018 The neural monitoring of visceral inputs, rather than attention, accounts for first-person perspective in conscious vision. Cortex **102**, 139–149. (10.1016/j.cortex.2017.05.019) 28651745

[68] Block N. 2023 The border between seeing and thinking. New York, NY: Oxford University Press.

[69] Berridge KC, Kringelbach ML. 2015 Pleasure systems in the brain. Neuron **86**, 646–664. (10.1016/j.neuron.2015.02.018) 25950633 PMC4425246

[70] Goodheart C. 1975 Problems of monetary management: the UK experience. In Inflation, depression, and economic policy in the west (ed. AS Courakis), p. 116. Totowa, NJ: Barnes and Noble Books.

[71] Prum RO. 2018 The evolution of beauty: how Darwin’s forgotten theory of mate choice shapes the animal world and us. New York, NY: Doubleday.

